# New Appliance

**Published:** 1867-10

**Authors:** 


*New Appliance.*—Dr. Henry Clark of this city has recently invented and brought to our notice a simple appliance, to be used in connection with the Inhaler, by which the administration of Nitrous Oxide Gas is greatly facilitated. It consists of a small square bag made of sheet rubber, capable of being partially inflated with air, and thereby forming a cushion which, by pressure with the fingers, is readily adapted to the surface of the face about the nose and mouth,

It is attached to the nozzle of the inhaler in the same manner as the simple sheet or disk of rubber cloth—by means of a hole near to the centre, through which the mouth-piece passes—and is pressed about the mouth and nostrils by two of the fingers of the left hand, while the other fingers of the same hand support the inhaling tube.

It is very simple and yet very serviceable.

